# Assessing the Efficacy of an App-Based Method of Family Planning: The Dot Study Protocol

**DOI:** 10.2196/resprot.6886

**Published:** 2017-01-18

**Authors:** Rebecca G Simmons, Dominick C Shattuck, Victoria H Jennings

**Affiliations:** ^1^ Institute for Reproductive Health Georgetown University Washington, DC United States

**Keywords:** fertility, fertility awareness, family planning, contraception, natural family planning, reproductive health, mHealth, mobile health apps, study protocol

## Abstract

**Background:**

Some 222 million women worldwide have unmet needs for contraception; they want to avoid pregnancy, but are not using a contraceptive method, primarily because of concerns about side effects associated with most available methods. Expanding contraceptive options—particularly fertility awareness options that provide women with information about which days during their menstrual cycles they are likely to become pregnant if they have unprotected intercourse—has the potential to reduce unmet need. Making these methods available to women through their mobile phones can facilitate access. Indeed, many fertility awareness applications have been developed for smartphones, some of which are digital platforms for existing methods, requiring women to enter information about fertility signs such as basal body temperature and cervical secretions. Others are algorithms based on (unexplained) calculations of the fertile period of the menstrual cycle. Considering particularly this latter (largely untested) group, it is critical that these apps be subject to the same rigorous research as other contraceptive methods. Dynamic Optimal Timing, available via the Dot app as a free download for iPhone and Android devices, is one such method and the only one that has published the algorithm that forms its basis. It combines historical cycle data with a woman’s own personal cycle history, continuing to accrue this information over time to identify her fertile period. While Dot has a theoretical failure rate of only 3 in 100 for preventing pregnancy with perfect use, its effectiveness in typical use has yet to be determined.

**Objective:**

The study objective is to assess both perfect and typical use to determine the efficacy of the Dot app for pregnancy prevention.

**Methods:**

To determine actual use efficacy, the Institute for Reproductive Health is partnering with Cycle Technologies, which developed the Dot app, to conduct a prospective efficacy trial, following 1200 women over the course of 13 menstrual cycles to assess pregnancy status over time. This paper outlines the protocol for this efficacy trial, following the Standard Protocol Items: Recommendations for Intervention Trials checklist, to provide an overview of the rationale, methodology, and analysis plan. Participants will be asked to provide daily sexual history data and periodically answer surveys administered through a call center or directly on their phone.

**Results:**

Funding for the study was provided in 2013 under the United States Agency for International Development Fertility Awareness for Community Transformation project. Recruitment for the study will begin in January of 2017. The study is expected to last approximately 18 months, depending on recruitment. Findings on the study’s primary outcomes are expected to be finalized by September 2018.

**Conclusions:**

Reproducibility and transparency, important aspects of all research, are particularly critical in developing new approaches to research design. This protocol outlines the first study to prospectively test both the efficacy (correct use) and effectiveness (actual use) of a pregnancy prevention app. This protocol and the processes it describes reflect the dynamic integration of mobile technologies, a call center, and Health Insurance Portability and Accountability Act–compliant study procedures. Future fertility app studies can build on our approaches to develop methodologies that can contribute to the evidence base around app-based methods of contraception.

**ClinicalTrial:**

ClinicalTrials.gov NCT02833922; https://clinicaltrials.gov/ct2/show/NCT02833922 (Archived be WebCite at http://www.webcitation.org/6nDkr0e76)

## Introduction

### Background

Current family planning options are not fully meeting the needs of women or men. Worldwide, some 222 million women want to avoid pregnancy but are not using family planning because of real or perceived side effects of methods or inaccurate perceptions of pregnancy risk at different points during their menstrual cycle or during breastfeeding [1]. The Family Planning 2020 (FP2020) goal of reaching 120 million additional women with family planning by 2020 is at its midpoint; new solutions to address unmet need are crucial to meeting this important milestone. Easy-to-use, affordable, fertility awareness-based methods, which can be made available through a variety of communication technologies, could fill a portion of this need.

Access to mobile phones, including smartphones, is increasing exponentially across the globe. According to the 2015 Ericsson Mobility Report, 2.6 billion people—25% of the world’s population—currently have a smartphone. It is estimated that by 2020 6.1 billion people—70% of the world’s population—will own a smartphone and that the vast majority (80%) of new smartphone subscriptions will come from Asia, the Pacific, the Middle East, and Africa [2]. In response to such large and diverse user bases, hundreds of thousands of smartphone apps have been developed and deployed, including more than 100,000 apps specifically focused on health issues [3]. Mobile phone apps have been shown to contribute to a range of positive health outcomes among people being treated for chronic conditions by providing information on frequently asked questions, reminders of appointments, medications, etc [4,5]. For family planning, smartphone apps also can provide timely information about specific contraceptive methods (how to use them, side effects, where to find services) and remind users about such actions as taking a pill or going for an injection [6].

However, fertility awareness-based methods, which require a woman to track her menstrual cycles and/or fertility symptoms but do not require a commodity or interaction with a provider, are the only methods that can be accessed entirely through a mobile app.

With these new technologies come new challenges. Currently, more than 1000 smartphone apps have been developed that focus on women’s menstrual cycles. The majority of these apps are designed simply to track cycles or to assist in planning a pregnancy. Most are not appropriate for prevention of pregnancy, although, alarmingly, there is evidence that women are using these apps for this purpose [7].

It appears that many apps model cycle lengths with a normal distribution and then estimate the day of ovulation by fixing the luteal phase to be 13 or 14 days, with days around ovulation identified as fertile. There are a number of drawbacks to such an approach when used for pregnancy prevention. These normative models are insensitive to unusual cycle lengths and may be less accurate when few observations are available. There are additional problems with the assumption of the luteal phase length as constant, since from a biological perspective, this is untrue. Most importantly, this approach does not incorporate uncertainty inherent in estimating the day of ovulation.

There are few apps that are appropriate for pregnancy prevention. A recent study by Duane et al [8] found that only 6 apps that claimed to be appropriate for pregnancy prevention could correctly identify the fertile window. The majority of these accurate apps are simply electronic platforms for existing, scientifically validated fertility awareness methods (FAMs), such as the sympto-thermal method or the Creighton-Billings method. For users of these methods, apps can represent a helpful mechanism allowing for quicker, more immediate entry of events (such as cervical secretions and basal body temperature) and may allow for data aggregation over time. However, because the majority of these methods are reliant on user interpretation, use of such apps is recommended in conjunction with appropriate training by a facilitator certified in a particular FAM method.

A very small number of apps make claims as stand-alone methods of contraception that essentially live within the app itself. These apps combine women’s personal fertility data (eg, period start date, basal body temperature) with proprietary algorithms in order to estimate times of high and low fertility within the menstrual cycle. Thus far, the existing literature on these apps has been limited to 3 studies.

The first study, assessing algorithm development and theoretical efficacy, was conducted on the Dot app in conjunction with the app developer, Cycle Technologies. The Dynamic Optimal Timing (DOT) algorithm for the Dot app was developed as a method of family planning that could be deployed on a mobile app platform without the need for any other assistive technology such as a basal body temperature thermometer. DOT’s predictions are based on Bayesian statistical calculations using pooled datasets of approximately 9000 cycles from several fertility studies to identify a woman’s fertile window [9]. Using the Dot app, women only need to record their first day of menses each cycle. When a woman downloads the app, she indicates whether she is using it to avoid pregnancy, achieve pregnancy, or simply track her cycles. The information the app provides her depends on her purpose for using it. For women who are avoiding pregnancy, Dot flags high risk days as those days with estimated probabilities of pregnancy above 1% to 2.25%, depending on the number of cycles she has entered [9]. As more data are collected, DOT appropriately updates these estimates, modifying her fertile window using her personalized cycle history. A description of the approach and an estimate of its efficacy (which found an estimated failure rate of less than 3 pregnancies per 100 women years of exposure with correct use) was published in 2016 [9]. The authors point to the need for a prospective study to determine actual perfect and typical use efficacy for pregnancy avoidance.

The other 2 studies were conducted on the Natural Cycles mobile app. Natural Cycles, which uses a proprietary algorithm described as being based on quantum physics, calculates fertility using basal body temperature (taken by the user on an external thermometer) and date of menstruation. The investigators found a 0.05% probability that the app would provide a false safe “green” and calculated typical use Pearl Index rate at 7 per 100 women years in a retrospective study [10]. As the authors acknowledge, the study was limited by its design, short time-frame, incomplete information about sexual intercourse frequency, and inability to verify all data entered into the app [10], reflecting the complexities of conducting an efficacy study on an app-based contraceptive method.

As more app-based methods debut in the mobile health, or mHealth, world, there is a growing need to establish standards of practice by which these methods can be evaluated. Prospective trials, which follow women over time to establish perfect and typical use rates, have long been the gold standard of contraceptive development [11]. However, unlike contraceptive devices or pills, app-based methods pose new opportunities and challenges in conducting such trials. Mobile technology expands opportunities to collect data in real time, potentially increasing the accuracy of self-report. Additionally, recruitment for efficacy trials using mobile technology can potentially capture broader geographies, as anyone who uses a particular mobile device could potentially be recruited, which may result in increased generalizability of study results. However, the challenges of identifying, recruiting, and retaining participant populations through mobile studies are well documented in the mHealth literature [12-15]. Challenges may be exacerbated when the study subject matter is around sensitive information such as sexual activity.

This paper details the protocol of a study assessing the effectiveness and efficacy of the Dot app in preventing pregnancy in reproductive-aged women in the United States. This trial is unique for several reasons. First, it is the first prospective efficacy trial conducted on an app-based method of family planning. Secondly, the deployment of this trial involves the development of a research enhancement to the app itself, which is activated when participants are consented into the study. Finally, the majority of data, including study-specific sexual activity and survey data, are collected through the app. In sharing this protocol, we attempt to outline our efforts to capitalize on some of the opportunities and address some of the challenges of conducting research via mobile apps. Our interest is to provide ongoing information about the development of this study, enhance transparency, and increase communication and dissemination around best practices for mobile research.

### Aims and Objectives

The aim of this efficacy study is to assess the Dot app as a new method of app-based contraception. The objectives for this study include (1) obtaining perfect and typical use pregnancy rates for users of Dot; (2) understanding how user interaction with the method is influenced by aspects such as demographics, social support, relationship support, and fertility awareness; and (3) assessing user preferences and best practices for conducting mobile contraceptive research.

## Methods

### Overview

The format of this efficacy protocol adheres to Standard Protocol Items: Recommendations for Intervention Trials (SPIRIT) guidelines [16].

This prospective, longitudinal, nonrandomized trial will be conducted on a cohort of women in the United States who download Dot on their Android phones with the objective of preventing pregnancy. While we would have preferred a randomized design, this was not possible. There is no other app with an established efficacy to which we could make reasonable comparison, either because no such study has been done or because behavioral components of using other apps are different from using Dot in ways that are likely to affect the characteristics of users and study retention. Even the CycleBeads app, which requires the same user inputs as the Dot app (period start date), is only appropriate for women with a more restricted cycle length range and variability. Thus, we are conducting a nonrandomized study. This study received ethical approval from the Georgetown University’s Institutional Review Board in May 2016 and is registered at ClinicalTrials.gov (NCT02833922).

**Figure 1:**
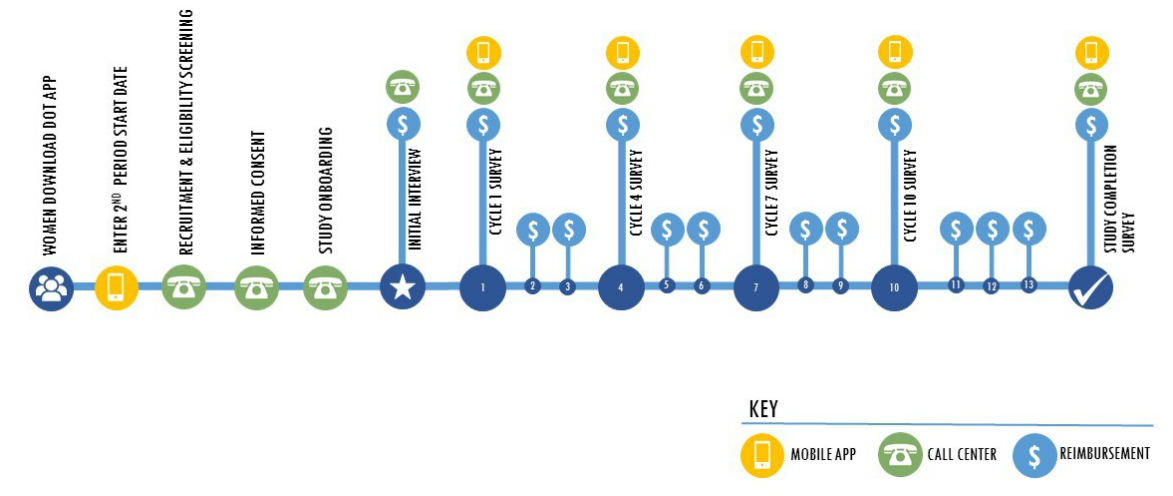
Dot study timeline.

In all other respects, we followed the Trussell and Kost guidelines for contraceptive efficacy studies [11]. Data collection, participant enrollment, and pregnancy definition are all influenced by their recommendations. Their guidelines also affect the way we will analyze the data, assessing both perfect and typical use, using life-table analysis (in additional to establishing a Pearl index), and observing women for up to 13 cycles of use. The approach has been adapted to meet the requirements of an app-based fertility awareness method. As there is virtually no experience conducting an app-based efficacy study for a method women access on their own prior to being recruited for the study, we will also attempt to minimize loss to follow-up by engaging women over time via the app and through communication with a call center. We will count pregnancies as reported by the woman, inquiring about her pregnancy status as soon as she fails to enter her period start date at the expected time to minimize undercounting pregnancies. Pregnancy results will be reported via a completed home pregnancy test kit, provided by mail, to the study team by women who believe they are or might be pregnant. While ideally we would enter women into the study the day they begin using Dot, it is not possible logistically, due to the structure of the app (a woman can download and begin using Dot on any day of her cycle by entering the day her most recent period started).Because they need to enter the study at the beginning of a cycle (to ensure they are not pregnant prior to entering the study and to obtain a complete history of sexual intercourse throughout the cycle), women will be recruited when they enter their second period start date. A brief visual illustration of the study timeline can be seen in Figure 1.

### Recruitment

Study participants will be initially recruited from the pool of women who choose to download the app to an Android phone to prevent pregnancy, as determined when they select “prevent pregnancy” as their mode of use. Upon the entry of their second period start date, women who have chosen to use Dot for pregnancy prevention will receive a pop-up message within the Dot app notifying them of the study and asking them if they are interested in participating.

### Eligibility Criteria

To be eligible to use the app to prevent pregnancy, participants must meet the following criteria:

- Self-reported usual cycle length of 20 to 40 days with less than 10 days variation
- Have not used hormonal contraceptives during their 3 most recent cycles

To be eligible for the study, participants must, in addition to meeting the Dot eligibility criteria

- Be between the ages of 18 and 39 years at the time of admission and live in the United States
- Be potentially at risk of pregnancy (ie, sexually active with a male partner)
- Be willing to install a password or biometric protection on their app for security purposes

Breastfeeding women can be admitted into the study upon meeting all other criteria if they have delivered at least 6 months ago and had at least 3 menstrual cycles postpartum. Women who do not meet one or more of these criteria will be excluded from the study.

### Screening

Upon receiving the initial recruitment message, users who are interested in participating will be provided with a prescreening questionnaire within the app. If women meet these prescreening criteria, they will be contacted by a call center representative, who will conduct a full eligibility screening.

### Consent

The call center study representative will review the informed consent document with potential participants. Women will also have the informed consent document available on their phones. Upon reviewing the consent, women will be asked to verbally consent and to electronically consent via the app.

### Study Onboarding

Women who are consented into the study will be informed that the research enhancement component of the app will be activated on their phone. Study staff will review data collection processes and will collect some baseline demographic data from the study participant. This will allow us to begin building a profile of our study population and to analyze the data to highlight similarities and differences among participants with different characteristics.

### Data Collection Methods

As part of the study, participants will be asked to provide daily sexual history information (whether they had intercourse on a particular day and if so, whether they also used a barrier method or emergency contraception) and their period start dates. They also will be asked to complete 4 brief surveys that will be distributed throughout the course of the study. Sexual history and period start date information will be collected exclusively through the app, but participants will have the choice to self-complete the surveys within the app or to be contacted by a study representative who can administer them.

### Exit Procedures

There are several circumstances under which participants may be exited from the study. Women whose cycles no longer meet the necessary criteria to use Dot (eg, experience short [<20 days], long [>40 days], or highly variable cycle lengths) will be exited from the study. Women who no longer wish to participate in the study or to use Dot will also be exited. Upon exiting the study, the research enhancement feature in the app will be disabled and all study elements removed. Participants will retain all cycle history data that they have entered into the app while participating in the study.

### Pregnancy Procedures

On the 41st day of a cycle, participants who have not yet entered a new period start date will receive a pop-up within the app asking them to either (1) enter a new date, (2) confirm that they are experiencing a long cycle, (3) confirm that they no longer wish to participate in the study, or (4) confirm that they think they may be pregnant. If a participant believes she might be pregnant, she will be directed to contact the study representative within 48 hours. Study representatives will send 2 (EPT brand) pregnancy tests to participants to confirm pregnancy. If the first pregnancy test is negative, participants will be instructed to wait an additional 5 days and take the second pregnancy test. Both pregnancy tests will be sent back to the study center in a prepaid mailing envelope for confirmation, and women will be exited from the study. Alternately, women can also contact the study representative if they had unprotected sex and believe they might be pregnant, which will again trigger the pregnancy exit protocol.

### Outcome Measures

Pregnancy status is the primary outcome measure. Because the study intends to provide both a perfect use and typical use failure rate, it is critical that for all pregnancies we determine whether it occurred as a result of intercourse on a day Dot identifies as fertile or infertile and if the intercourse resulting in pregnancy was protected (by use of a barrier method) or unprotected. We will review the intercourse data entered by the participant for the cycle in which pregnancy occurred and annotate pregnancies as resulting from (1) unprotected intercourse on a day Dot identified as fertile, (2) unprotected intercourse on a day Dot identified as nonfertile with no intercourse or only protected intercourse on a day identified as fertile, (3) protected intercourse on a day Dot identified as fertile with no intercourse on an identified fertile day, or (4) protected intercourse on a day Dot identified as nonfertile with no intercourse on a day identified as fertile. While there is a small possibility that pregnancy could result from protected or unprotected intercourse on a nonfertile day in a cycle with only protected intercourse on a fertile day, it is not possible to determine with the data we will be able to collect for this study.

The main secondary outcome is discontinuation of use of the Dot app for pregnancy prevention (to become pregnant, because participants are no longer sexually active, or because they prefer another method of family planning) or discontinuation from the study (because their cycle length is outside the range or variability covered by Dot, they chooses to leave the study, or they are lost to follow-up).

### Data Management, Forms, Entry, Transmission, and Editing

**Figure 2:**
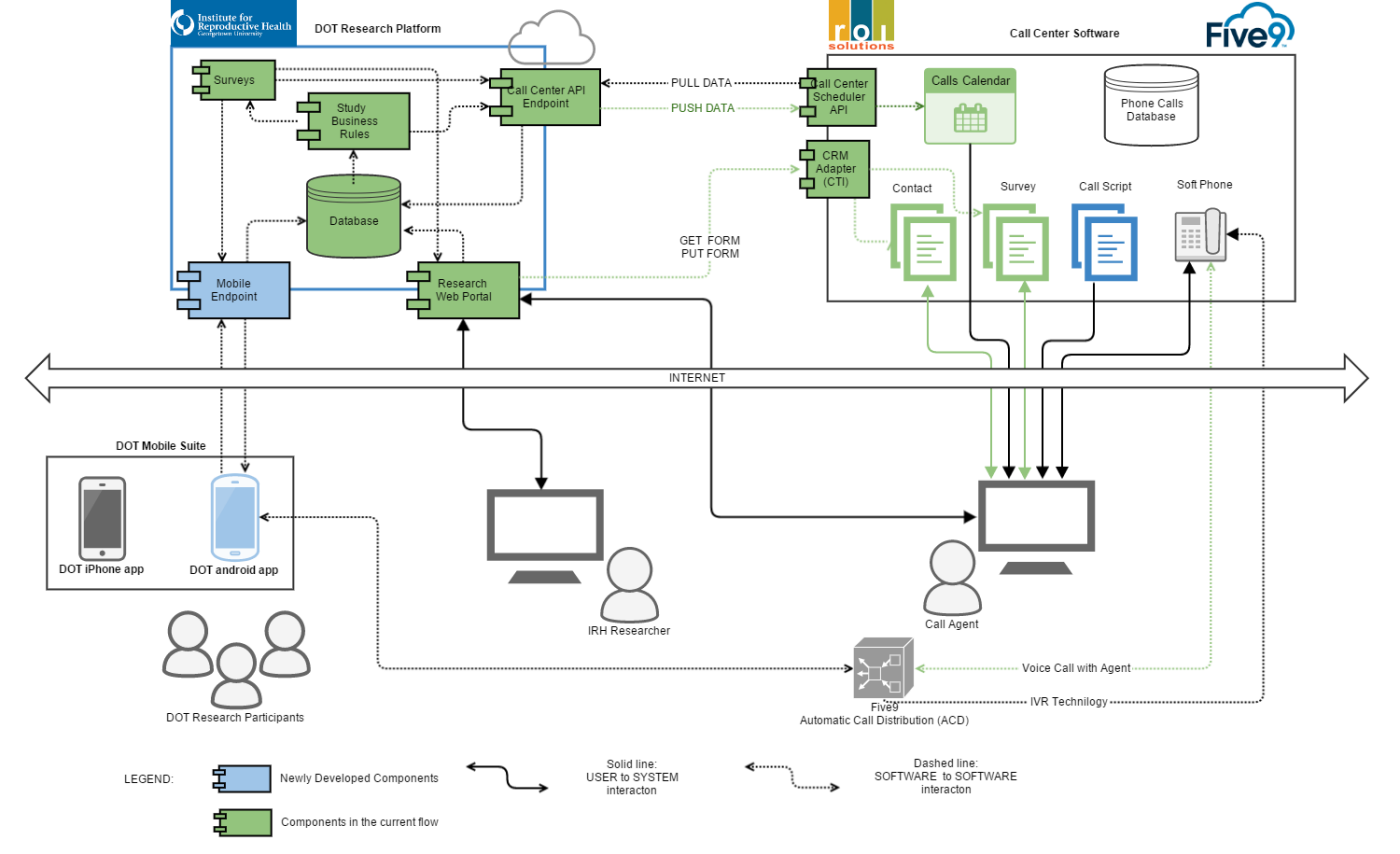
Illustration of cloud-based architecture for the Dot study.

The technical architecture for the Dot study will use cloud services provided by Amazon Web Service (AWS). The Institute for Reproductive Health (IRH) technical solution will be hosted within the northern Virginia region, as it is one of the largest AWS regions, as well as within the same geographical area as Georgetown University and the IRH offices. AWS provides a simple and streamlined way to access servers, storage, and databases over the Internet. There are no physical study site locations where participants will interact with study staff; study data will be collected through the app transmitted via transport layer security (TLS) and encrypted and stored in AWS DynamoDB, which is a scalable, highly available storage solution from AWS. Figure 2 illustrates the cloud-based architecture which will be used to collect and host research data. Furthermore, range checks along with a data dictionary will be used to enforce the integrity of the data. Participants will be able to modify their daily sexual history and period start date data within the current menstrual cycle. Once the menstrual cycle has been completed, the data will be considered locked for the study purposes.

### Data Discrepancy Inquiries and Reports

Data will be reviewed periodically and checked for consistency and to identify any missing data. A number of standard analytic reports on various aspects, such as recruitment, study status, survey completion, and other relevant data points, will be developed and run through the system throughout the duration of the study.

### Security and Backup

All data in transit will be secured by https/TLS. Data at rest will be secured using a variety of encryption methods, including Amazon Elastic Block Store encryption and Relational Database Service encryption. The AWS ecosystem consists of distributed and fully redundant data centers strategically located throughout the United States. The AWS storage solution will deliver highly scalable and reliable cloud storage for backup and recovery. Data backups have been configured based on the research design and information needs; however they will be performed using a combination of incremental and full backups.

### Description of Hardware

Each computer is configured per Georgetown University’s university information system security standards with passwords settings, secure imaging, and computer firewall software. The desktop systems are Dell Latitudes E5470/E7250 and Lenovo X1 Carbon Thinkpads operating on a Windows 7 enterprise. Symantec Endpoint protection and Malwarebytes Anti-Malware Premiums will be used as the main personal computer anti-virus software.

### Confidentiality

Several steps will be taken throughout the course of the study to ensure participant confidentiality. Women who agree to participate must agree to set a passcode for the app so that the app and study data are protected. All study data will be pushed, pulled, and stored in accordance with or exceeding Health Insurance Portability and Accountability Act compliance standards set forth by Georgetown University.

### Sample Size

Our estimated sample size, given 80% power, 95% confidence levels, and an assumed relative risk of 3, is 432 participants [17]. However, our sample size decision-making was also influenced by considerations for study attrition, which is a significant concern in longitudinal, web-based research [10,18]. We factored in previously identified variables negatively affecting attrition, such as marital status [19], younger-aged study participants [20], and research on a mobile platform [21], which are known attributes of our proposed research cohort. Given these factors, we see a need to enroll a significantly larger number of women to ensure that a sufficient number of women complete 13 cycles of study participation and to allow for the calculation of valid efficacy and effectiveness rates. Based on the rate of discontinuation in the Standard Days Method efficacy study (54.4%) and the higher potential for attrition in this particular study, we plan to overpower the proposed study by recruiting up to 2000 women and enrolling 1200 women. Quarterly, the IRH study team will evaluate the discontinuation rate and determine whether new or additional recruitment efforts for the study will be deployed [22]. We will also examine characteristics of participants who exit our study to identify potential threats to external and internal study validity.

### Outcome Analyses

To address questions related to efficacy/effectiveness, we will use a prospective, single-arm design following women from their first full cycle of Dot use, as identified when women enter the start of their second period and continuing for up to 13 cycles (14th period start date). We will apply single-decrement multicensoring life tables to calculate Dot efficacy (probability of nonpregnancy status) and rates of continuation (women-months of method use). Censoring cycles will facilitate the use of more cycles in outcome analysis, while accounting for issues of missing data or loss to follow-up [23].

Dot efficacy will be assessed by typical use (correct plus incorrect use resulting in pregnancy) and method failure (correct use resulting in pregnancy) pregnancy rates. The unit of analysis will be the cycle and pregnancies recorded by cycle of Dot use. Both life tables and a Pearl Index (number of pregnancies per 100 women-years) will be calculated. A 95% CI will be calculated and the SE computed [24,25].

Women’s cycles will be censored if

- They choose to discontinue from the study or are lost to follow up
- They report an experience that restricts their provision of data (eg, loss of cell phone, moving to an area with no coverage)

Periodically, we will examine the pattern of discontinuation and censored data to identify potential biases in study participation. In accordance with standard life table analyses assumptions, we will assume that there are no changes in participation over time, that data are simply missing, that the experience of individuals who are lost to follow-up is the same as the experience of those who are followed, and that both pregnancy and study discontinuation of the participants occurs uniformly within the interval [26].

A series of descriptive statistics will be calculated to contextualize the questions outlined above regarding user profiles, ability to correctly use the method and the app, previous method use, and user satisfaction.

## Results

Funding for the study was provided in 2013 under the United States Agency for International Development (USAID) Fertility Awareness for Community Transformation (FACT) project. Recruitment for the study will begin in January 2017. The study is expected to last approximately 18 months, depending on recruitment. Findings on the study’s primary outcomes are expected to be finalized by September 2018.

## Discussion

The main aims of the Dot efficacy study are to estimate the efficacy and effectiveness of the Dot app for pregnancy prevention. The findings from this trial will represent the first prospective efficacy trial conducted on an app-based method of family planning. This is an essential step in developing a research base to support the use of these methods as part of the existing contraceptive method mix and identifying lessons for establishing best practices to guide future research on similar apps. A stronger evidence base for fertility awareness apps will serve both the global family planning community and consumers who wish to use these methods but have little concrete information on their efficacy.

In addition to our main objectives, our study will also attempt to answer additional questions around several relevant topics to both reproductive health and to mHealth research. The real-time nature of data collection methods via mobile phone can contribute to ongoing research questions about fertility awareness methods in general, including important questions around sexual activity decisions during the fertile time, changes in couple communication over the course of fertility awareness method use, and changing reproductive goals. Additionally, we intend to assess several components of mHealth research more broadly, specifically around participant recruitment and retention questions such as research participant engagement, gamification of research, and data questions, such as which methods of data collection participants prefer (phone/instant messaging/in-app surveys). Our findings will contribute to the broader mHealth research agenda assessing these important questions.

Our study involves the creation of a supplemental research enhancement that overlays and is activated within an existing commercial app. Collaboration between research institutes and commercial app developers represents a potentially exciting opportunity in mHealth. Yet, such collaborations require significant deliberations around how to develop and implement quality research studies in a way that maintains app fidelity and doesn’t inhibit user experience. We anticipate that our experiences and findings will also provide insights into the opportunities and challenges of collaboration and can provide recommendations for future research/developer partnerships.

## Abbreviations

AWS: Amazon Web Service
DOT: Dynamic Optimal Timing
FACT: Fertility Awareness for Community Transformation
FAM: fertility awareness method
FP2020: Family Planning 2020
IRH: Institute for Reproductive Health
SPIRIT: Standard Protocol Items: Recommendations for Intervention Trials
TLS: transport layer security
USAID: United States Agency for International Development

## Appendix

Multimedia Appendix 1 CONSORT-EHEALTH checklist V1.6.2 [27].
