# Conflict of Interest Addendum: Assessing the Efficacy of an App-Based Method of Family Planning: The Dot Study Protocol

**DOI:** 10.2196/resprot.8829

**Published:** 2018-03-16

**Authors:** Rebecca G Simmons, Dominick C Shattuck, Victoria H Jennings

**Affiliations:** ^1^ Institute for Reproductive Health Georgetown University Washington, DC United States

The authors of “Assessing the Efficacy of an App-Based Method of Family Planning: The Dot Study Protocol” (JMIR Res Protoc 2017;6(1):e5) have a previously undisclosed competing interest as follows:

The product under investigation (the Dynamic Optional Timing [DOT] app) is the property of Cycle Technologies, Inc, a for-profit corporation based in Washington, DC. The CEO of Cycle Technologies is Leslie Heyer (née Jennings), who is the daughter of Victoria Jennings, one of the co-authors of this article. The efficacy study on DOT uses funds from a research grant awarded to the Institute for Reproductive Health at Georgetown. Cycle Technologies Inc or their employees do not receive any licensing fees, honoraria, or financial contributions related to the study.

The corrected article will appear in the online version of the paper on the JMIR website on March 16, 2018, together with the publication of this correction notice. Because this was made after submission to PubMed or PubMed Central and other full-text repositories, the corrected article also has been re-submitted to those repositories.

